# Age-Related Vitamin D Deficiency Is Associated with Reduced Macular Ganglion Cell Complex: A Cross-Sectional High-Definition Optical Coherence Tomography Study

**DOI:** 10.1371/journal.pone.0130879

**Published:** 2015-06-19

**Authors:** Mathieu Uro, Olivier Beauchet, Mehdi Cherif, Alix Graffe, Dan Milea, Cedric Annweiler

**Affiliations:** 1 Department of Ophthalmology, Angers University Hospital, Angers, France; 2 Department of Neuroscience, Division of Geriatric Medicine, Angers University Hospital, University Memory Clinic of Angers; UPRES EA 4638, University of Angers, UNAM, Angers, France; 3 Singapore National Eye Centre, Singapore; 4 Singapore Eye Research Institute, Singapore; 5 Duke-NUS, Neuroscience and Behavioral Diseases, Singapore; 6 Robarts Research Institute, Department of Medical Biophysics, Schulich School of Medicine and Dentistry, the University of Western Ontario, London, Ontario, Canada; Charité University Medicine Berlin, GERMANY

## Abstract

**Background:**

Vitamin D deficiency is associated with smaller volume of optic chiasm in older adults, indicating a possible loss of the visual axons and their cellular bodies. Our objective was to determine whether vitamin D deficiency in older adults is associated with reduced thickness of the ganglion cell complex(GCC) and of the retinal nerve fibre layer(RNFL), as measured with high-definition optical coherence tomography(HD-OCT).

**Methods:**

Eighty-five French older community-dwellers without open-angle glaucoma and patent age-related macular degeneration(mean, 71.1±4.7years; 45.9%female) from the GAIT study were separated into 2 groups according to serum 25OHD level(i.e., deficient≤25nmol/L or sufficient>25nmol/L). Measurements of GCC and RNFL thickness were performed using HD-OCT. Age, gender, body mass index, number of comorbidities, dementia, functional autonomy, intracranial volume, visual acuity, serum calcium concentration and season of testing were considered as potential confounders.

**Results:**

Mean serum 25OHD concentration was 58.4±26.8nmol/L. Mean logMAR visual acuity was 0.03±0.06. Mean visual field mean deviation was -1.25±2.29dB. Patients with vitamin D deficiency(n=11) had a reduced mean GCC thickness compared to those without vitamin D deficiency(72.1±7.4μm versus 77.5±7.5μm, P=0.028). There was no difference of the mean RNFL thickness in these two groups(P=0.133). After adjustment for potential confounders, vitamin D deficiency was associated with reduced GCC thickness(ß=-5.12, P=0.048) but not RNFL thickness(ß=-9.98, P=0.061). Specifically, vitamin D deficiency correlated with the superior medial GCC area(P=0.017) and superior temporal GCC area(P=0.010).

**Conclusions:**

Vitamin D deficiency in older patients is associated with reduced mean GCC thickness, which can represent an early stage of optic nerve damage, prior to RNFL loss.

## Introduction

Besides its classical function of bone metabolism regulation, vitamin D exhibits multiple biological targets mediated by its nuclear hormone receptor, the Vitamin D Receptor (VDR) [1,2]. In particular, by binding to the VDRs present in the endothelial retina cells [2,3] and in the neurons and glial cells of the brain [4], vitamin D influences a number of physiological and protective processes in the central nervous system (CNS), including the regulation of neurotrophins and neuromediators, as well as anti-inflammatory, antiangiogenic and antioxidant effects [3,4]. Consistently, vitamin D status has demonstrated links with brain health but also with eye health and function [5–11]. Age-related lower serum 25-hydroxyvitamin D (25OHD) concentrations have been associated with worse visual acuity among older adults [7]. The reason for this association is the purpose of much research. Parallel to the possibility of an involvement of vitamin D in macular health [8], with vitamin D deficiency promoting age-related macular degeneration (AMD) [9–13], other 'beyond-the-eye' explanations are also investigated. In particular, a previous work by our team has reported that vitamin D deficiency is associated with smaller volume of optic chiasm in older adults [14], indicating possible loss of visual axons and retinal ganglion cells associated with vitamin D deficiency. This assumption is consistent with the neurosteroid effects of vitamin D reported in the CNS including in the optic nerve [3,4], but also with the recent finding of an association between vitamin D deficiency and primary open-angle glaucoma (POAG) [15]. Interestingly, POAG has been previously associated with dementia [16,17], another condition in which vitamin D deficiency may play a role [6,18,19]. To date, it remains unclear whether vitamin D deficiency has an impact on the optic nerve structure. Our purpose here was to determine in older adults whether vitamin D deficiency was associated with reduced thickness of the ganglion cell complex (GCC) and of the retinal nerve fibre layer (RNFL) measured with high-definition optical coherence tomography (HD-OCT).

## Material and Methods

### Participants

Between 30 November 2009 and 20 December 2012, 91 older community-dwellers were followed in the Memory Clinic of Angers University Hospital, France, for a subjective memory complaint, and were recruited into the Gait and Alzheimer Interactions Tracking (GAIT) study (ClinicalTrials.gov number, NCT01315717). The GAIT study is an observational cross-sectional study designed to examine gait in older community-dwellers reporting subjective memory complaint. The sampling and data collection procedures have been described elsewhere in detail [20]. In summary, subjective memory complaint was documented using the Subjective Memory Complaints Questionnaire [21], and the main exclusion criteria were age below 60 years, Mini-Mental State Examination (MMSE) score <10 [22], inability to walk independently, history of stroke, history of any acute medical illness within the past 3 months, current delirium, severe depression, and inability to understand or answer the study questionnaires. For the present analysis, we only included patients without past or current ophthalmic conditions such as POAG or patent AMD. Thus, six participants who were diagnosed with previously unknown POAG were excluded from the study. Finally, 85 participants were included in this analysis.

In addition to a full medical examination and blood tests for vitamin D, calcium and albumin concentrations, all included participants underwent an ophthalmic evaluation, including best corrected visual acuity, pachymetry, standard automated perimetry (Program 30–2 of the Humphrey field analyzer, Carl Zeiss, Dublin, CA) with SITA-standard algorithm, fundoscopy, retinal fundus color imaging and HD-OCT.

### Dependent variables: GCC and RNFL thickness measurements with OCT

GCC and RNFL were determined automatically and analyzed using spectral-domain Cirrus HD-OCT model 4000 (software version 6.0; Carl Zeiss; Meditec, Dublin, CA). The pupil was not dilated. GCC thickness was isolated from a standard macular cube of 6 x 6 mm (512 x 128 scans), with global and sectoral measurements (i.e., superior nasal, superior medial, superior temporal, inferior nasal, inferior medial, inferior temporal). RNFL thickness was measured circularly at 3.4mm of the papillary center. Global and sectoral thickness measures (i.e., nasal, superior, temporal, inferior) were noted. Retinal segmentation was automatically performed by the program and immediately controlled by an experienced orthoptist who was blinded from participants’ vitamin D status. Measurements were retried in case of misalignment. Arbitrarily, all measures were performed on the right eye of each participant, except for 2 participants with an epiretinal membrane in the right eye.

### Explanatory variable: Serum vitamin D deficiency

Venous blood was collected from resting participants. Serum 25OHD concentration, an effective indicator of vitamin D status [1,2], was measured by radioimmunoassay (DiaSorin corp., Stillwater, MN). Intra- and interassay precisions were respectively 5.2% and 11.3%. Vitamin D deficiency was defined *a priori* using the consensual 25OHD threshold of ≤25 nmol/L (to convert to ng/mL, divide by 2.496) i.e., deep and chronic hypovitaminosis D most likely to generate adverse health events [1,2,23]. All measurements were performed locally at the University Hospital of Angers, France.

### Covariables

The best corrected visual acuity was measured using decimal Monoyer charts and converted into logMAR units for statistical analysis purposes. After fundoscopy, images of the retinal fundus were systematically taken via non-mydriatic fundus photography and reexamined post-hoc by an experienced ophthalmologist. Evaluation of comorbidities (i.e., diseases lasting at least 3 months and running a course with minimal change, whatever the etiology) was based on self-report and medical record. All participants in the study had a cognitive assessment at the time of inclusion. Dementia was diagnosed using the consensus criteria of the Diagnostic and Statistical Manual of Mental Disorders, fourth edition [24]. Functional autonomy was assessed using the Instrumental Activities of Daily Living (IADL) score [25], with a score ranging from 0 to 4, best. The body mass index (BMI) was calculated as: [weight (kg) / height^2^ (m^2^)]. Weight was measured with a beam balance scale, and height with a height gauge. The intracranial volume, which was reported to possibly influence the OCT measures [26], was measured in cm^3^ to account for interindividual differences in the size of cranial cavity that could explain differences in intracranial su-volumes and thicknesses independent of vitamin D status. The intracranial volume was obtained from 3D T_1_-weighted MP-RAGE images obtained from 1.5 Tesla MRI scanner (Magnetom Avanto, Siemens Medical Solutions, Erlangen, Germany) (acquisition matrix = 256x256x144, FOV = 240mm x 240mm x 187mm, TE/TR/TI = 4.07ms/2170ms/1100ms) and analyzed using FreeSurfer (v5.1.0), a set of tools that automatically segments, labels and quantifies brain tissue volumes (http://surfer.nmr.mgh.harvard.edu/) [27]. The season of evaluation was recorded as follows: spring from March 21 to June 20, summer from June 21 to September 20, fall from September 21 to December 20, winter from December 21 to March 20. Finally, the serum concentration of calcium was measured using automated standard laboratory methods at the University Hospital of Angers, France. Because of the high prevalence of hypoalbuminemia in older adults, calcium values were corrected according to the formula: [corrected calcium value = Ca + 0.02 (46-albumin)].

Age, gender, BMI, number of comorbidities, dementia, IADL score, intracranial volume, visual acuity, serum calcium and season of testing were considered as potential confounders in our analysis.

### Statistical analysis

The participants’ characteristics were summarized using means and standard deviations or frequencies and percentages, as appropriate. As the number of observations was higher than 40, no transform was applied [28]. Firstly, comparisons of participants' characteristics according to vitamin D status (i.e., deficiency of not) were performed using Student's *t*-test or the Chi-square test, as appropriate. Secondly, the mean difference of the GCC and RNFL thickness was calculated between participants with vitamin D deficiency and those with no vitamin D deficiency. Thirdly, multiple linear regressions were used to examine the associations of vitamin D deficiency (explanatory variable) with the GCC and RNFL thickness (dependent quantitative variables), while adjusting for potential confounders. Separate analyses were performed for this purpose. Finally, we examined the correlation between vitamin D deficiency and the thickness of the subregions of the GCC and RNFL. P-values <0.05 were considered significant. All statistics were performed using SPSS (v19.0; SPSS, Inc., Chicago, IL) and RevMan (v5.1, Nordic Cochrane Centre, Copenhagen, Denmark).

### Ethics

Participants participating in the study were included after having given their written informed consent for research. The study was conducted in accordance with the ethical standards set forth in the Helsinki Declaration (1983), and was approved by the University of Angers Ethical Review Committee (CPP Ouest II—2009–12).

## Results

**Fig 1:**
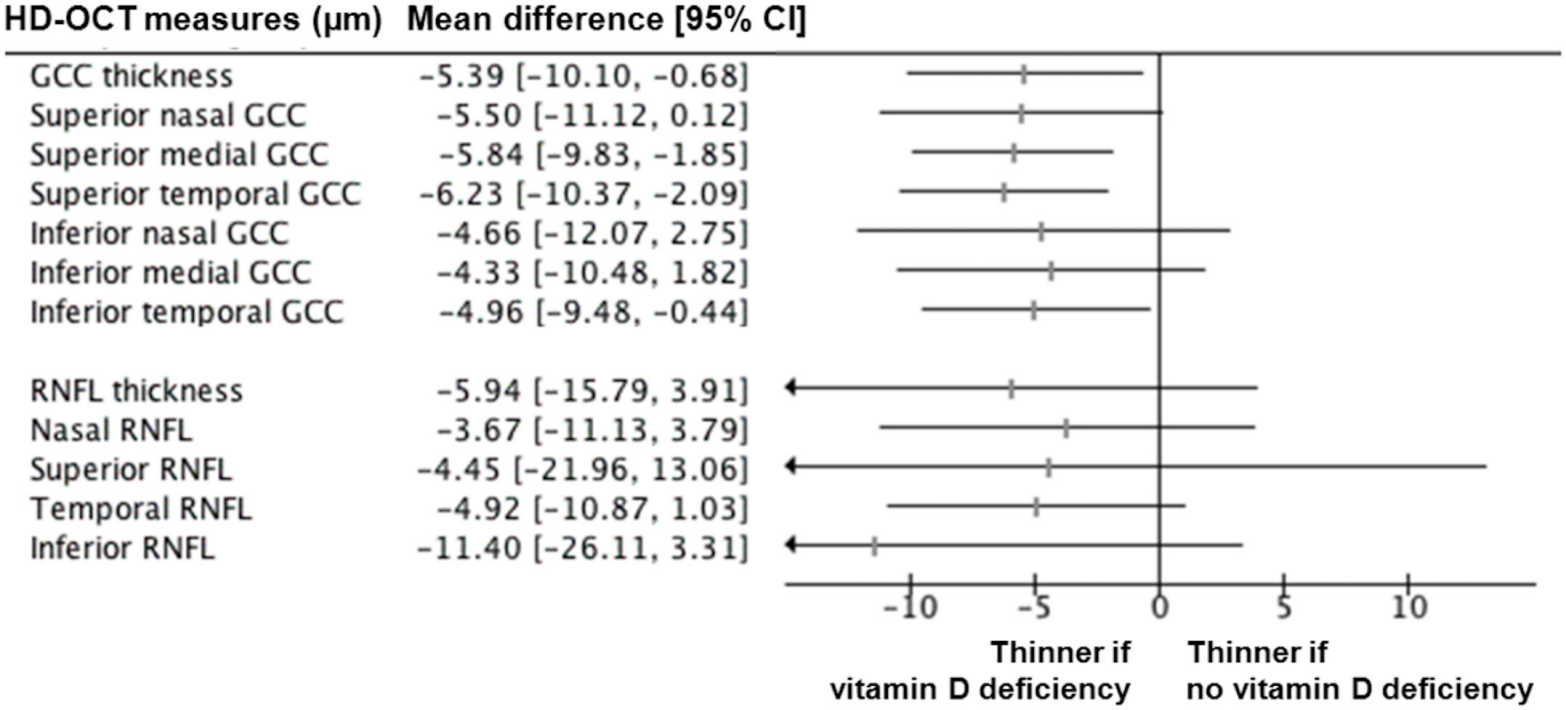
Forest plot for the mean difference of ganglion cell complex (GCC) and retinal nerve fibre layer (RNFL) thickness according to vitamin D deficiency (i.e., serum 25-hydroxyvitamin D ≤ 25 nmol/L). Horizontal lines correspond to the 95% confidence interval (CI). The vertical line corresponds to a mean difference of 0.00, equivalent to no between-group difference.

Among 85 older participants with no patent ophthalmic disease who were included in this analysis (mean age, 71.1±4.7 years; 45.9% female; 100% Caucasian), the mean serum 25OHD concentration was 58.44±26.80 nmol/L. Eleven participants (12.9%) had vitamin D deficiency. The mean logMAR visual acuity was excellent, i.e. 0.03±0.06 and the mean visual field mean deviation was -1.25±2.29 dB (Table 1), with no difference between those with and those without vitamin D deficiency (P = 0.689 and P = 0.818, respectively). Overall, 82/85 patients had vision better than 20/25 in both eyes. Among the included patients, no cornea or other anterior segment abnormalities were noted, except cataract, which was previously successfully operated in 13 of the included patients. Only 7 patients were diagnosed with mild cataract, with relatively preserved vision (higher than at least 20/50 on the right eye). Ten participants had asymptomatic macular drusen, but no patent AMD (P = 0.194 for between-group comparison). Only 2 participants had microcysts in the inner layer of the macula, with no between-group difference (P = 0.581). Similarly, there was no difference of macular thickness (P = 0.298). As indicated in Table 1, the participants with vitamin D deficiency had lower mean GCC thickness than those with 25OHD>25nmol/L (72.09±7.44μm versus 77.48±7.46μm, P = 0.028). The mean difference in thickness was -5.39μm [95% confidence interval (CI): -10.10;-0.68] (Fig 1). In contrast, the RNFL thickness did not differ according to the vitamin D status (respectively 84.18±16.07μm versus 90.12±11.47μm, P = 0.133). There were no between-group differences for the other characteristics (Table 1). As illustrated in Table 2, fully adjusted linear regression showed a significant association of vitamin D deficiency with the mean GCC thickness (ß = -5.12, P = 0.048), but not with the mean RNFL thickness (ß = -9.98, P = 0.061).

**Table 1:** Baseline characteristics of 85 participants by vitamin D status.

	Total cohort (n = 85)	Vitamin D deficiency*		P-value
		Yes (n = 11)	No (n = 74)	
**Demographical measures**				
Age, years	71.13±4.71	73.32±5.99	70.80±4.44	0.098
Female gender, n (%)	39 (45.9)	4 (36.4)	35 (47.3)	0.497
**Clinical measures**				
Body mass index, kg/m^2^	26.19±3.62	25.80±2.59	26.25±3.76	0.701
Number of comorbidities	2.28±1.78	2.64±0.92	2.23±1.88	0.484
Dementia, n (%)	5 (5.9)	0 (0.0)	5 (6.8)	0.374
IADL score, /4	3.76±0.53	3.64±0.67	3.78±0.50	0.390
Intracranial volume, cm^3^	1517.65±170.27	1534.71±138.32	1515.07±175.57	0.764
**Ophthalmic findings**				
Visual acuity, logMAR	0.03±0.06	0.02±0.04	0.03±0.06	0.689
Visual field mean deviation, dB	-1.25±2.29	-1.40±1.74	-1.22±2.37	0.818
Drusen detection, n (%)	10 (11.8)	0 (0.0)	10 (13.5)	0.194
Macular microcysts detection, n(%)	2 (2.4)	0 (0.0)	2 (2.7)	0.581
Mean macular thickness, μm	277.45±13.28	273.55±14.27	278.04±13.13	0.298
Mean GCC thickness, μm	76.77±7.64	72.09±7.44	77.48±7.46	**0.028**
Mean RNFL thickness, μm	89.35±12.21	84.18±16.07	90.12±11.47	0.133
**Serum measures**				
25-hydroxyvitamin D, nmol/L	58.44±26.80	19.00±3.77	64.30±23.56	**<0.001**
Calcium, mmol/L	2.37±0.10	2.34±0.11	2.37±0.10	0.342
**Season**				0.619
Spring, n (%)	20 (23.5)	2 (18.2)	18 (24.3)	
Summer, n (%)	16 (18.8)	1 (9.1)	15 (20.3)	
Autumn, n (%)	47 (55.3)	8 (72.7)	39 (52.7)	
Winter, n (%)	2 (2.4)	0 (0.0)	2 (2.7)	

Data presented as mean±standard deviation when applicable. AMD: age-related macular degeneration; GCC: ganglion cell complex; IADL: Instrumental Activities of Daily Living; RNFL: retinal nerve fibre layer;

*: Serum 25-hydroxyvitamin D ≤ 25 nmol/L; P-values<0.05 indicated in bold.

**Table 2:** Fully adjusted linear regressions examining the association between vitamin D deficiency* (explanatory variable) and the GCC and RNFL thickness^†^ (dependent quantitative variables), adjusted for potential confounders (n = 85).

	High-definition Optical Coherence Tomography					
	GCC thickness			RNFL thickness		
	β	[95% CI]	P-value	β	[95% CI]	P-value
Vitamin D deficiency	-5.12	[-10.18;-0.06]	**0.048**	-9.98	[-20.46;-0.49]	0.061
Age	-0.58	[-1.03;-0.13]	**0.013**	-0.43	[-1.37;0.50]	0.356
Female gender	2.09	[-2.50;6.67]	0.365	1.37	[-8.08;10.82]	0.772
Body mass index	-0.51	[-1.01;0.00]	0.050	-0.24	[-1.29;0.80]	0.641
Number of comorbidities	-0.20	[-1.25;0.84]	0.696	-0.50	[-2.65;1.66]	0.645
Dementia	-4.95	[-13.11;3.21]	0.229	-8.99	[-25.88;7.90]	0.290
IADL score	-3.75	[-8.01;0.52]	0.084	-7.70	[-16.52;1.11]	0.085
Intracranial volume	0.00	[-0.01;0.01]	0.968	0.01	[-0.20;0.03]	0.592
Visual acuity	1.15	[-27.79;30.10]	0.998	-13.73	[-73.60;46.14]	0.647
Calcium	11.42	[-7.79;30.64]	0.238	-4.38	[-43.44;34.68]	0.823
Season	-0.87	[-3.02;1.28]	0.420	-1.26	[-5.63;3.11]	0.564

β: coefficient of regression corresponding to a change of thickness; CI: confidence interval; GCC: ganglion cell complex; IADL: Instrumental Activities of Daily Living; RNFL: retinal nerve fibre layer;

*: Serum 25-hydroxyvitamin D ≤ 25 nmol/L;

^†^: separate models used for each cognitive score; β significant (i.e., P-value<0.05) indicated in bold.

Table 3 reports the correlations between vitamin D deficiency and the thickness of the different sectors of the GCC and the RNFL. Vitamin D deficiency correlated negatively with the mean superior medial GCC (r = -0.26, P = 0.017) and superior temporal GCC (r = -0.28, P = 0.010), and there was a trend for a correlation with the superior nasal GCC (r = -0.21, P = 0.055). In contrast, vitamin D deficiency did not correlate with the inferior sectors of the GCC. Finally, there were no correlations between vitamin D deficiency and the different sectors of the RNFL (Table 3). Using serum 25OHD concentration as a quantitative variable, we found no significant correlation with neither the mean GCC thickness (r = 0.16, P = 0.142) nor the mean RNFL thickness (r = 0.15, P = 0.164).

**Table 3:** Correlation of vitamin D deficiency^†^ with the thickness of the different sectors of the ganglion cell complex (GCC), and with the thickness of the different sectors of the retinal nerve fibre layer (RNFL).

	**GCC**	**Superior nasal GCC**	**Superior medial GCC**	**Superior temporal GCC**	**Inferior nasal GCC**	**Inferior medial GCC**	**Inferior temporal GCC**
**Vitamin D deficiency** ^†^	-0.24*	-0.21	-0.26*	-0.28**	-0.18	-0.18	-0.18
	**RNFL**	**Nasal RNFL**	**Superior RNFL**	**Temporal RNFL**	**Inferior RNFL**		
**Vitamin D deficiency** ^†^	-0.16	-0.09	-0.09	-0.15	-0.18		

*: P<0.05 (2-tailed);

**: P≤0.01 (2-tailed);

***: P<0.001 (2-tailed);

^†^: Serum 25-hydroxyvitamin D ≤ 25 nmol/L

## Discussion

The main finding of this OCT study is that, irrespective of potential confounders, vitamin D deficiency was associated with reduced thickness of the mean GCC layer, and more specifically in its superior sectors. In contrast, there was no association between vitamin D deficiency and RNFL thickness.

To the best of our knowledge, this study is the first to assess and report such an association. This novel finding is consistent with recent literature reporting an association between vitamin D deficiency and structural abnormalities of the afferent visual pathways. In one prior study performed among 62 older adults (mean, 71.2±5.0 years; 45.2% female), vitamin D deficiency was associated with reduced macular thickness among older patients with no patent macular dysfunction (P = 0.001) [8]. Parallel, in another study, 20 independent community-dwelling older adults (mean, 74.3±6.3 years; 40.0% female) exhibited a direct association between serum 25OHD concentration and the volume of optic chiasm, with lower 25OHD relating to smaller optic chiasm [14]. The latter finding suggested the possibility of loss of the retinal ganglion cells and their axons associated vitamin D deficiency. Yet, only MRIs were performed in this latter study, and no detailed ophthalmologic evaluation was available. Thus, the results of the current study provide additional information. Specifically, we found that, in older adults without any known or clinically patent optic nerve condition, vitamin D deficiency was associated with GCC but not with RNFL thinning. Reduction of the macular retinal GCC, composed by the retinal ganglion cell layer and the inner plexiform layer, may be an early, initial manifestation of retinal ganglion cells loss. Indeed, the retinal ganglion cells are densest in the macula and form a stratified multi-cellular layer within the central area of the visual field. Loss of axons and/or retinal ganglion cell bodies in this region will result in thinning of the macular retinal ganglion cell layer, visible on HD-OCT. Thus, it has been suggested that thickness of the macula OCT and GCC complex could provide a better structural indicator of neuronal and/or axonal loss compared to the peripapillary RNFL scan in certain optic neuropathies [29]. Macular GCC measures may be better at detecting macular damage, while peripapillary RNFL measures would identify damage outside the macula [30]. Additionally, the high density of the retinal ganglion cells in this region, and the lack of vessels, which can play a confounding role in the peripapillary region, are important factors when measuring this region with OCT, as compared to the peripapillary region.

The mechanism linking vitamin D deficiency with loss of retinal ganglion cells is not clearly understood. Two approaches, which can be interconnected, are possible to explain such an association: 1) vitamin D deficiency has direct, specific consequences on intraocular structures. If the retinal ganglion cells were specifically susceptible to vitamin D deficiency, we would expect that their loss could cause a patent neurodegenerative optic neuropathy, at least in some severe, or longstanding cases. The neuronal loss in our study was probably affecting preferentially the macular region, and thus possibly detected at an early stage, before thinning of the RNFL and occurrence of a clinically patent optic neuropathy, such as POAG. Consistent with this assumption is the lack of visual impairment in the case of vitamin D deficiency in our sample (Table 1), although such an association has previously been reported [7]. A similar situation can be seen in other optic neuropathies, such as dominant optic atrophy, in which early retinal ganglion cell loss occurs in the macular region [31,32], followed later by RNFL thinning. Interestingly, vitamin D deficiency has been recently associated with POAG, a neurodegenerative optic neuropathy, among 6094 participants recruited in the Fifth Korean National Health and Nutrition Examination Survey [15]. Consistently, animal experimentation has reported that vitamin D eye drops resulted in a reduction of intraocular pressure by 20% in non-human primates, mainly by increasing the uveoscleral outflow [33]. None of our patients had POAG or high intraocular pressure, which were from the start necessary exclusion criteria, in order to reduce potential confounders. However, we cannot rule out that some of the participants with reduced GCC thickness and vitamin D deficiency in our study may subsequently evolve towards a form of glaucoma, not necessary associated with high intraocular pressure. A prospective follow-up of these groups of patients is currently ongoing at our institution. 2) A second speculative approach is to consider that vitamin D deficiency is linked to generalized neurodegeration, and that our original ophthalmic findings in asymptomatic patients would represent a possibly early manifestation, or even biomarker of this global degeneration. The tight interconnections between brain and retina have prompted several authors to consider the retina as a privileged window to the brain, allowing early diagnosis and monitoring of neurodegenerative diseases [34]. As a related example, it is nowadays accepted that vitamin D deficiency is associated with all-cause dementia and Alzheimer disease [18,19], conditions in which retinal ganglion cell loss and RNFL thinning can be detected with OCT [35,36]. More specifically, thinning of the macular retinal GCC was recently found in patients with Alzheimer’s disease (AD) [37]. Various neuroprotective effects of vitamin D in the CNS and the eye may explain these associations. In vitro, vitamin D increases the synthesis of neurotrophic agents such as the Nerve Growth Factor (NGF) and the Glial cell line-Derived Neurotrophic Factor (GDNF), and regulates neuronal differenciation and maturation [3,4]. It also accelerates neuronal growth in a dose-dependent way in rat hippocampal cell cultures [38]. Parallel, the anti-inflammatory properties of vitamin D may be involved as there is evidence for a pivotal role of vitamin D in the immune system [39,40] by modulating effect on the immune cells that produce vitamin D and express the VDR. Finally, the antioxidant properties of vitamin D against oxidative stress of reactive oxygen and nitrogen species in the central nervous system [41] should also be mentioned.

The finding that vitamin D deficiency is associated with reduced GCC thickness has interesting potential clinical implications. Indeed, even if there was no correlation in our study between GCC thickness and serum 25OHD concentration used as a quantitative variable, it is of note that providing a result in terms of linear correlation—in other words, reporting a change in GCC thickness related to a change of 1 nmol/L of serum 25OHD—has only poor significance for clinical practice compared to showing an association between vitamin D deficiency and thinner GCC. To the best of our knowledge, there are no clear reference values for a ‘clinically relevant change’ in GCC thickness. In our study, we found a significant decrease of 5.39 μm (7.0%) in GCC thickness when comparing vitamin D sufficiency with vitamin D deficiency (Fig 1). Such estimates may help to justify, plan, evaluate, and compare in the future the effectiveness of interventions aiming at preventing optic neuropathy with vitamin D supplements that would utilize change in GCC thickness as an outcome measure.

The strengths of our study include the originality of the research question on a highly common condition in older adults, the standardized collection of data from a single research center, and the detailed description of the participants' characteristics allowing the use of regression models to measure adjusted associations. Regardless, a number of limitations should be acknowledged. First, the study cohort was restricted to relatively healthy community-dwelling older participants who might be unrepresentative of the population of all seniors. Of note, our results are consistent with previous epidemiological cohorts since, beyond our main finding, we also reported that the GCC thickness depended on advancing age, which is consensual [42] and strengthens the relevance of our studied sample. Nevertheless, it is noticeable that lower 25OHD concentrations are also frequently encountered in children, adolescents and young adults [43]. Thus additional studies are needed to test the association between vitamin D deficiency and thickness of the optic pathways at the different stages of life. Second, the cohort was limited to 85 participants, which may have exposed to lack of statistical power. Third, although we excluded participants with POAG and patent AMD, a small proportion of participants with other ocular conditions, such as asymptomatic drusen, were still included. Fourth, at present, our study is cross-sectional, which limits conclusions regarding causality. Despite these limitations, we were able to show a 7-percent reduction in GCC thickness, but no reduced RNFL, with vitamin D deficiency among older adults with normal or subnormal vision. We propose that vitamin D deficiency may be associated at early stages with infraclinical, limited neuronal loss in the macular region, more easily detectable with spectral OCT. At later neurodegenerative stages, additional thinning of RNFL could in turn be detected. Further prospective studies are needed to clarify whether older adults with vitamin D deficiency are more likely to experience GCC and RNFL thinning than those with normal vitamin D status, and whether the correction of vitamin D deficiency could improve, or prevent, this process.
